# Tunnel motion: Pupil dilations to optic flow within illusory dark holes

**DOI:** 10.1177/03010066241270493

**Published:** 2024-08-28

**Authors:** Bruno Laeng, Shoaib Nabil, Akiyoshi Kitaoka

**Affiliations:** University of Oslo, Norway; RITMO Centre for Interdisciplinary Studies in Rhythm, Time and Motion, University of Oslo, Norway; University of Oslo, Norway; 1948 University of Sussex, UK; Ritsumeikan University, Japan

**Keywords:** illusory motion, optic flow, darkness, pupillometry

## Abstract

We showed to the same observers both dynamic and static 2D patterns that can both evoke distinctive perceptions of motion or optic flow, as if moving in a tunnel or into a dark hole. At all times pupil diameters were monitored with an infrared eye tracker. We found a converging set of results indicating stronger pupil dilations to expansive growth of shapes or optic flows evoking a forward motion into a dark tunnel. Multiple regression analyses showed that the pupil responses to the illusory expanding black holes of static patterns were predicted by the individuals’ pupil response to optic flows showing spiraling motion or “free fall” into a black hole. Also, individuals’ pupil responses to spiraling motion into dark tunnels predicted the individuals’ sense of illusory expansion with the static, illusory expanding, dark holes. This correspondence across individuals between their pupil responses to both dynamic and static, illusory expanding, holes suggests that these percepts reflect a common perceptual mechanism, deriving motion from 2D scenes, and that the observers’ pupil adjustments reflect the direction and strength of motion they perceive and the expected outcome of an increase in darkness.

Two-dimensional dynamic patterns that show continuous and systematic changes in screen pixels’ brightness, can simulate the perception of optic flow and therefore of motion inside a virtual “hole” or “tunnel.” These realistic motion effects are well known from digital animations and films, where motion within a virtual 3D space can be evoked by a systematic radial flow in local brightness levels. In some of these displays, despite the changes in pixels’ brightness are very local, often happening only at the contours of a pattern (Gregory & Heard, 1983), like in the reversed phi movement effect (Anstis, 1970; Kitaoka, 2006) or four-stroke apparent motion (Mather & Murdoch, 1999), the perceived motion is illusorily attributed to the whole pattern and, in some instances, also evoking a motion inside a virtual “tunnel.” Remarkably, some entirely static displays can evoke perception of the patterns as if they were expanding or moving (Laeng et al., 2022), despite no change or flux in pixels’ luminance (Figure 1). Simply the presence of smooth brightness gradients or blur can, in most observers, evoke a sense of flow from within the pattern, as if watching some digital animation or a dynamic *gif,* instead of the actual, static, *jpg* image. In all cases, observers get a distinctive impression of optic flow that can evoke in the observer the perception of moving inside a tunnel, towards a central dark hole.

**Figure 1.**
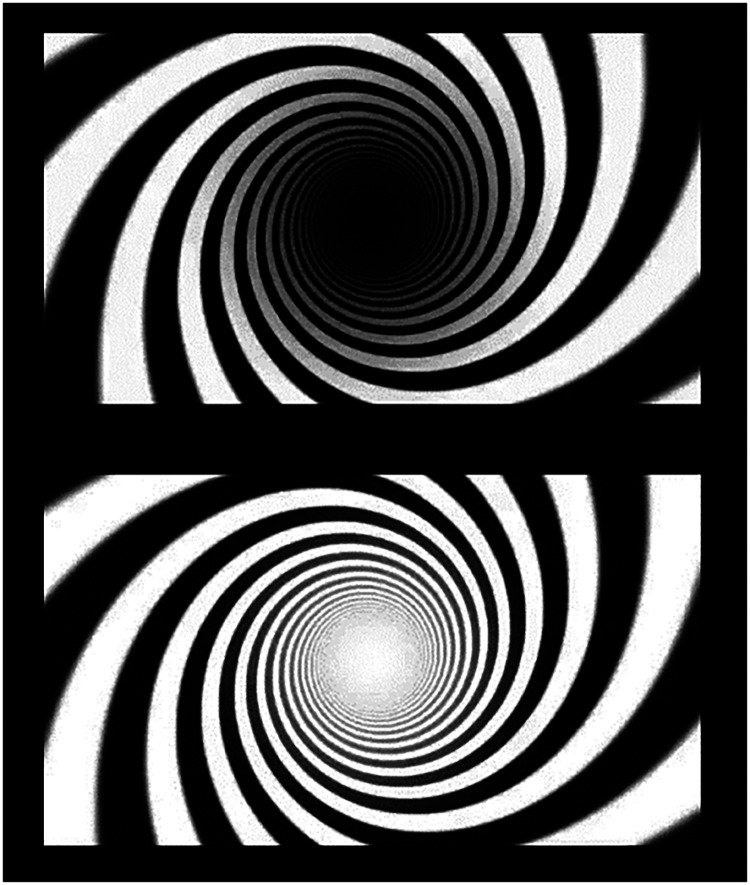
Snapshots of dynamic stimuli that showed spiraling motion towards a dark or bright hole.

The stimuli below, in Figures 1–3 but not 4, when seen full screen on an LCD screen, tend to evoke an impression of moving or even falling into a three-dimensional space or void. The motion perception with these two-dimensional visual stimuli appears very similar (i.e., flow, expansion), but only the dynamic patterns consist of a flux in pixels’ brightness that emulates the sensory changes on the retinal surface (e.g., the outflow of points according to an ordered structure) that would occur when an observer is really moving inside a tunnel. However, the static patterns only suggest that possibility, based on the presence of “smear” gradients around the dark center, which can imply motion (Changizi, 2009, chapter 3). Yet, all these stimuli are inherently ambiguous, though in different degrees, since in neither case observers are exposed to anything else than two-dimensional, physically flat, images; in fact, there are really no tunnels, or holes, and hence no motion of the observers or of objects within a 3D world.

**Figure 2.**
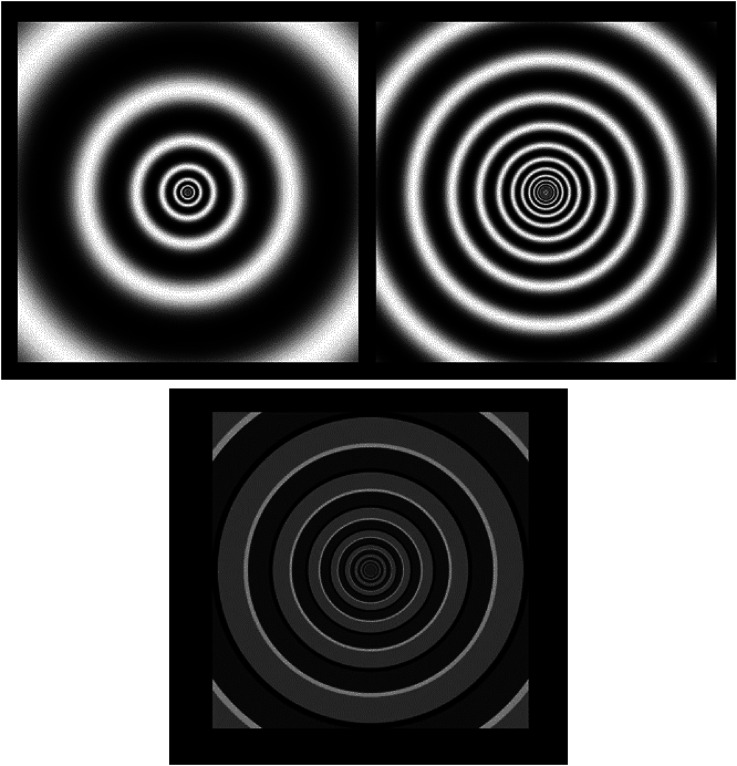
Snapshots of dynamic stimuli with different expansion speeds (top left: high speed; top right: low speed) that show apparent motion in a tunnel, the bottom snapshot shows an example of a reversed phi dynamic stimulus.

**Figure 3.**
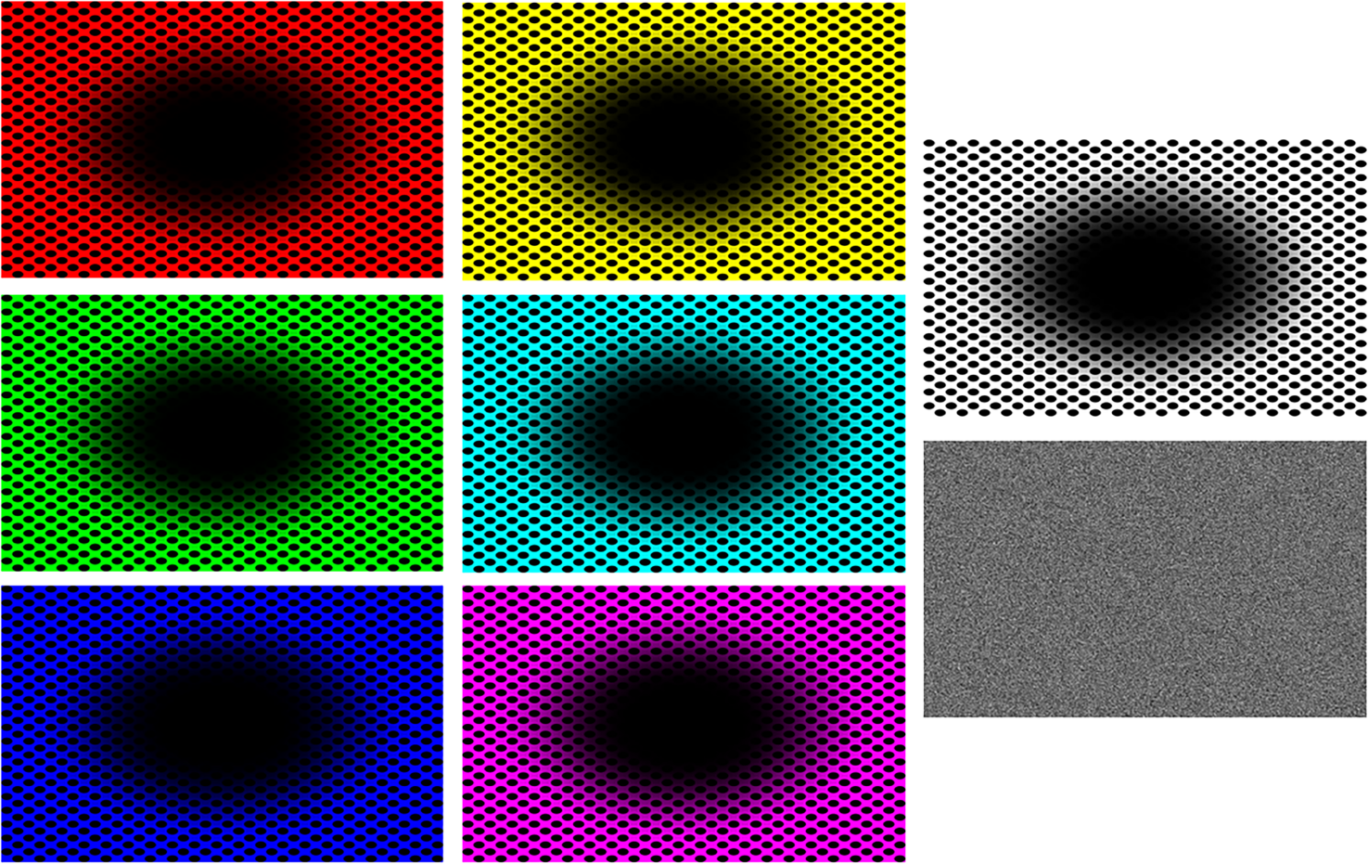
Illustrations of the static patterns showing the illusorily expanding “dark holes” as well as an example of the baseline (by shuffling all pixels) for the black and white pattern.

Several scholars have argued that the basic problem of perception is an intrinsic ambiguity of sensory stimulation and that observers’ brains have evolved to construe percepts by inferring their causes and achieve the most likely or “optimal” account of the external world (e.g., Brown & Friston, 2012; Kersten et al., 2004). Regarding “light,” either as a source or as a reflection over object surfaces, its perception is not straightforwardly related to the original physical parameters (Purves et al., 2014).

Instead, the brightness attributed to elements of the visual world are constructed from sensory input by exploiting learned, statistical, regularities in visual experience, coupled with the behavioral consequences of settling for a percept instead of another (Purves et al., 2004b). Behavioral success provides the feedback or “pragmatic validity” that the resulting percept fits the external world, whatever that reality may ultimately be (Purves et al., 2004a). Hence, there is a continuum between perception and optical illusions, since they all derive from the same powerful constraints that allow to resolve perceptual ambiguities and, in crucial cases, to successfully anticipate how visual experience is likely to change or what is probable to happen in the next moment (Changizi et al., 2008).

The present study applies the psychophysiological method of pupillometry with the systematic study of illusory optic flow. In a previous study (Laeng et al., 2022), we showed that when observers viewed static patterns that tended to evoke a perception of illusory expansion of a central dark hole, like the top image in Figure 1, observers’ eye pupils dilated proportionally to everyone's phenomenological report (by ratings on a Likert scale) about the experienced strength of the illusory flow. We interpreted these findings as evidence that the illusory flow of darkness from the center of these stimuli triggered an anticipatory response of the pupil to prepare for a likely decrease in brightness due to the impression of moving into a dark space. This account is also consistent with a proposal that the illusory perceptions of the majority of known optical illusions reflect the brain's predictive construal of the next moment under 3D perceptual interpretations of the 2D pattern (Changizi et al., 2008). In the present case, all stimuli implied an expansive dark hole and, in turn, the observer's forward motion.

Only a few previous studies have investigated the pupil response to illusory motion. Beukema et al. (2017) showed 2D geometrical patterns that evoked an illusory motion of a peripheral drift on the plane of the screen and compared the pupil response to similar patterns that did not “move”; they found greater pupil dilation for the illusion than for the control pattern. In another study (Castellotti et al., 2021), pupil responses were measured while observers looked at real motion (in videoclips) or either illusory or implied motion in still photographs. All types of stimuli caused a pupil dilation compared to control patterns, though with different amplitudes and onsets; the really moving stimuli dilated the pupil more than all other types of stimuli. It is likely that these differences depended on the strength of the motion percept; indeed, static images with implied motion enhanced by blur, motion streaks, and speed lines, triggered stronger pupil responses than images with simple “freeze action.” Hence, the pupil response signaled the level of attention and arousal evoked by the stimuli. In general, illusions are pleasant and exciting to watch (Stevanov et al., 2012) and really-moving stimuli strongly engage primary visual areas and self-motion engages other, possibly specialized, cortical areas (like within the entorhinal cortex; Campbell & Giocomo, 2018). Remarkably, illusory motion can activate the specialized motion areas of the cortex (V5 or MT; e.g., Zeki et al., 1993). Hence, these previous studies likely indicate that motion, whether real or illusory, captures attention and this is reflected in the pupil, as an index of mental effort; for example, the larger the set of moving stimuli that are attended, in a multiple object tracking task, the more the pupil dilates (Alnæs et al., 2014). However, all above studies (except that of Laeng et al., 2022) did not address the question of whether the pupil dilation response was related to an evoked impression of expansive motion or of an impending change in luminance, which is what the present study aims to clarify using different types of dynamic and static illusory-expansive optic flows.

Hence, we showed on a computer screen the same “dark hole” static patterns of our previous study (Laeng et al., 2022) that successfully evoked, in most of those participants and in variable degrees, the illusory perception of optic flow towards a dark central region. This part of the study can be considered a replication attempt. The novel aspects of the present study are that we compared, in the same participants, the pupil responses to the static illusory stimuli with those to dynamic illusory stimuli or animations of optic flow into virtual tunnels. Some of these dynamic stimuli evoked optic flow due to the continuous expansions of rings of different brightness within the pattern; other patterns had local brightness flux generating the so-called “four-stroke apparent motion effect” (Mather & Murdoch, 1999). Given that we posit a continuity of underlying mechanisms between perceiving real dynamic changes in luminance and the other illusory varieties, we would expect that the oculomotor responses to the static and dynamic stimuli would be strongly related at both the group and individual levels. In the specific case of optic flows with increasing darkness, whether the flow really occurs, or it is just illusory, we expect a dilation of the pupil as a sign of an optimal preparatory response. Note that, in the case of static illusory images, such a response would happen despite the “flow” is only illusory and brightness is constant; yet, even in the dynamic flow images that we present here, despite the presence of highly visible flux in local brightness (e.g., at the contour of rings), the images’ brightness remains unchanged globally and over time. Yet, we predict that the oculomotor responses will reflect the phenomenology that both types of image share: seeing expansive motion and, in turn, a virtual motion into a dark hole or tunnel. Note that if the physical light parameters stimulating the retina mattered most, the pupil diameters should either remain at constant levels or oscillate around a constant value while looking at these patterns. In contrast, if the preset account is correct and the phenomenology or perception itself influences the pupil response, then the pupil diameters should gradually dilate and do so proportionally to the perceived intensity of each expansive flow.

## Methods

### Participants

Forty-four participants (24 females, ages 21–45, mean age = 29 years; *SD *= 7.6) were recruited at the University of Oslo, agreeing to participate anonymously in a perceptual study. The participants were treated according to the declaration of Helsinki and the study was approved by the University's IRB. All participants had normal vision or corrected by contact lenses.

### Stimuli and Apparatus

**Figure 4.**
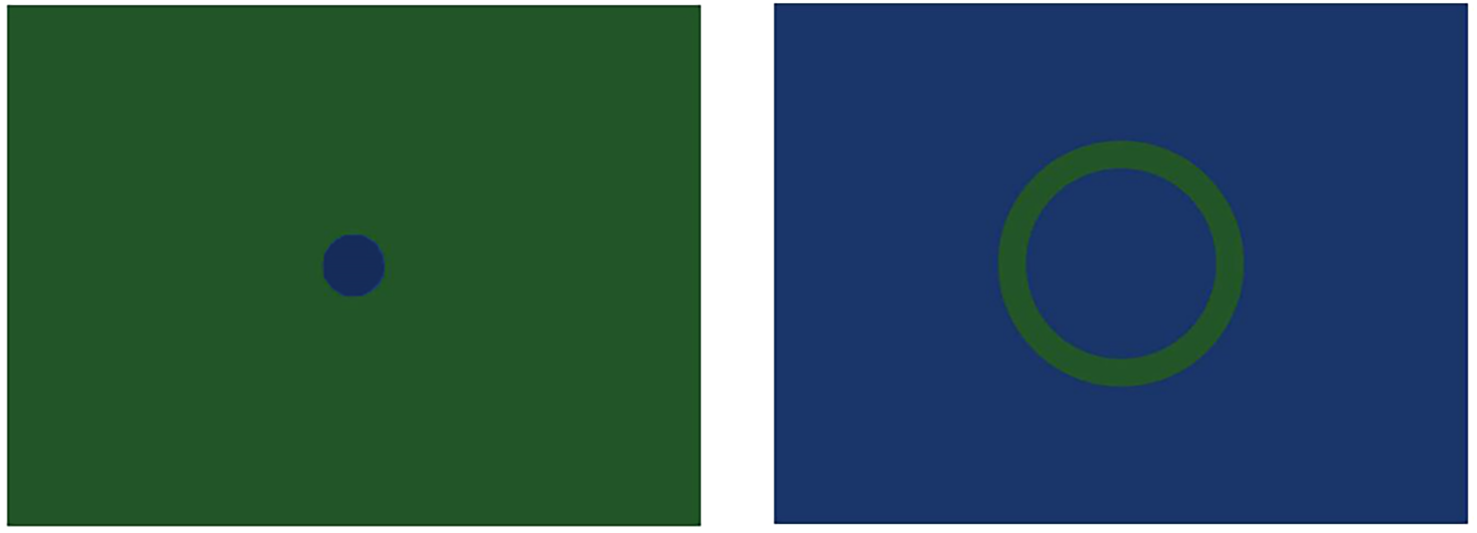
Snapshots of dynamic stimuli that showed expanding/contracting circles or rings (either blue on green or green on blue; all colors equiluminant).

All stimuli consisted in two-dimensional geometrical patterns that belonged to three types: (a) eight video animations (in *avi* format) showing optic flow patterns (circular or spiraling) where the observers had the perception of viewing tunnels (Figures 1 and 2), with either expanding or contracting motion, also evoking visual sensations of self-motion in depth, either forward (when expanding) or backward (when contracting). A pair of stimuli differed also in spatial frequency (i.e., width of the moving rings; Figure 2, top). Most of these video animations (Figure 1 shows snapshots; the animations are available here: https://figshare.com*,* doi: 10.6084/m9.figshare.25272886) were developed by Akiyoshi Kitaoka but the spiraling stimuli were downloaded from royalty-free stock photos websites (e.g., *depositphotos.com*, *vecteezy.com*). (b) seven static images that evoked the illusory perception of a central, expanding, dark hole (Figure 3); (c) eight video animations (in *avi* format) showing, blue/green-colored, rings or circles on an equiluminant green/blue background (Luminance = 52 in HSL), that either expanded or shrank over the surface of the screen, appearing clearly reduced in depth compared to the other animated images (Figure 4), and where observers had no sense of self-motion within a virtual space; additionally, one stimulus showed several gray circles or rings continuously moving (expanding/contracting), although this stimulus evoked weak or no impressions of motion in depth. The “dark holes” consisted of seven patterns (in *PNG* format), where the color background changed across stimuli, consisting of a central, elliptical, black-filled region on a background of equally spaced colored dots. All these patterns were developed by Akiyoshi Kitaoka and were also used in a previous study by the same authors of the present article. These stimuli are available here: https://figshare.com*,* doi: 10.6084/m9.figshare.25272991. Between the target stimuli, there were “baseline” images generated either by blurring the original videos (with a Gaussian blur function) or by randomly scrambling all the pixels of the static images. In both cases, the original patterns were not recognizable, but both the dynamic and static images were equiluminant to the subsequent, corresponding, pattern stimuli, adjusting for the same duration the pupils to the average luminance of the target stimuli.

### Procedure

Participants were seated 65 cm from the screen, with the head stabilized in a chinrest. At the beginning of each block, they completed a standard four-point calibration procedure. All stimuli were presented full screen for a fixed duration of 8 s and preceded by the baseline stimuli for the same duration. All stimuli were shown in a random order within two blocks, one with the static stimuli (seven trials, each stimulus shown once) and another with the dynamic stimuli (18 trials, each stimulus shown twice). There was a single session, lasting about half an hour, and the order of the blocks was counterbalanced across participants.

A Dell LCD monitor displayed the patterns full screen at a resolution of 1680 × 1050 pixels. Pupil diameters were obtained with an infrared SMI R.E.D. About 500 at a sampling rate of 60 Hz. Participants were instructed to simply look freely at the patterns on screen without closing the eyes. No rating of motion was required after each presentation of the animated videos since they all evoked a robust perception of motion either in 2D or in depth. Instead, in the second block with the dark holes, right after the presentation of a stimulus, each participant rated the amount of subjective (illusory) motion on a Likert scale (1–5), as done in our previous study (Laeng et al., 2022). The randomized pixels stimuli were presented right before each corresponding pattern and for the same duration of 8 s. The purpose of these “baselines” was to let the pupils adapt to the average brightness of the following stimulus, removing carry-over effects due to the brightness of a preceding pattern.

## Results

Using *BeGaze* software (SMI), we extracted each participant's expansion ratings for the dark holes and the pupil data in each trial of every block. Pupil data were based on pupil diameters (in mm) during fixations only, as computed by *BeGaze*, effectively excluding artifacts due to blinks or data obtained during saccades. For most analyses, we averaged pupil diameters in mm across all fixations that occurred during each 8 s period of viewing a stimulus. The pupillometry data files with the animations (*avi*) are available here: doi: 10.6084/m9.figshare.25272925, whereas the pupillometry data files with the static images (*png*) are available here: doi: 10.6084/m9.figshare.25272991

### Motion into Circular or Spiraling Tunnels

These stimuli evoked significantly different pupil changes between expanding and contracting motion. Since previous studies have shown that pupil diameters are nearly normally distributed (Mathôt et al., 2018), we run two separate, repeated-measures, analyses of variance with mean pupils (mm) as the dependent variable.

**Figure 5.**
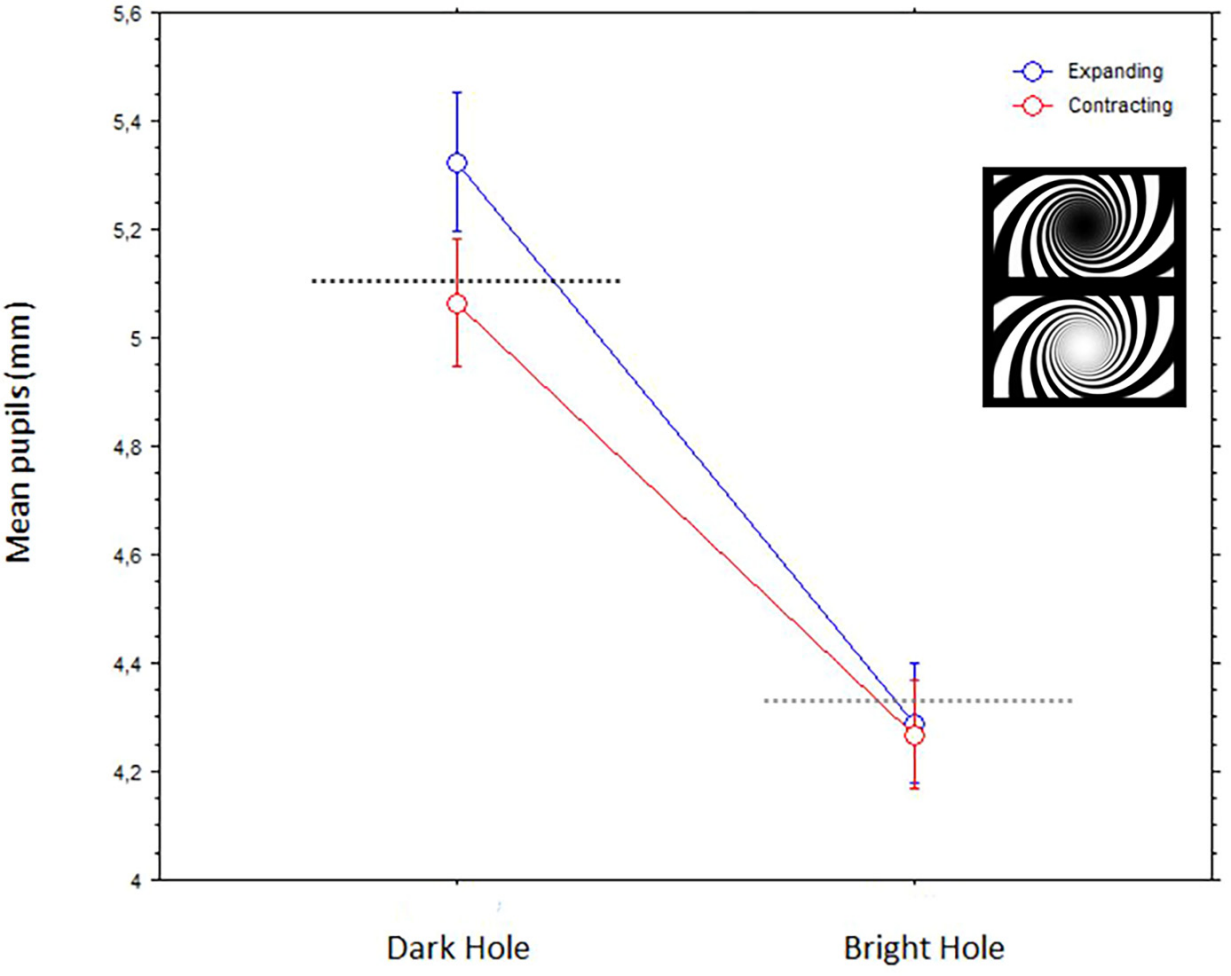
Mean pupils (bars are standard errors) to the dynamic spiraling tunnels with different directions of motion (expanding or contracting) and central holes (dark and bright).

In the analysis with the spiraling dynamic patterns, the within-subject factors were hole (dark/bright) and direction of motion (expanding, contracting). Both factors had significant main effects; direction of motion, F(1,42) = 10.7, *p* = .0022, and Hole: F(1,42) = 347.2, *p* < .0001. The two factors interacted, F(1,42) = 9.7, *p* = .0032. Figure 5 illustrates these results; remarkably the largest pupil dilation took place when viewing the dark spiraling tunnel expanding (or with forward motion).

**Figure 6.**
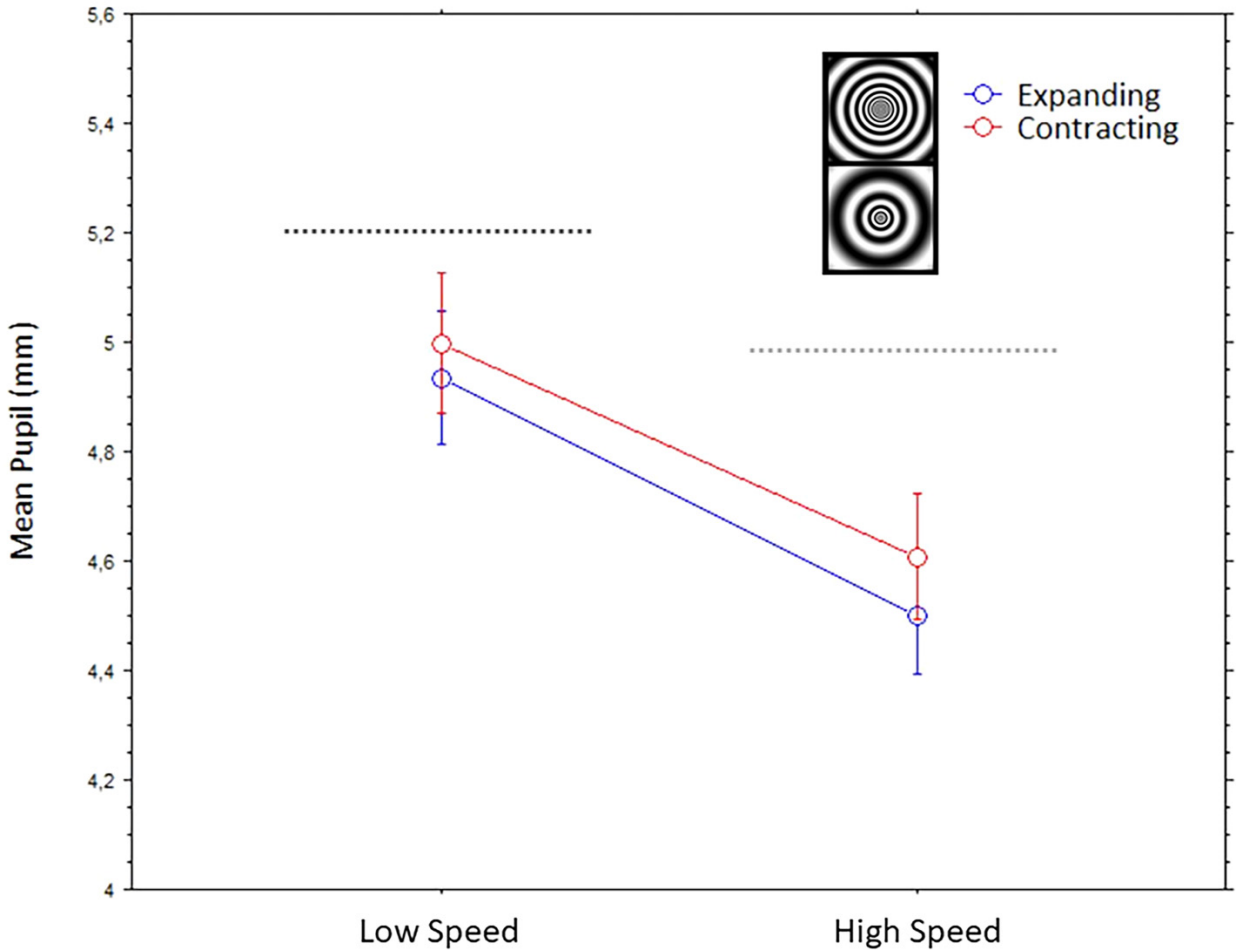
Mean pupils (bars are standard errors) to the dynamic circular tunnels with different directions of motion (expanding or contracting) and speed (high and low).

In the analysis with the tunnel-like stimuli, the within-subject factors were direction of motion (expanding, contracting) and speed (high speed, low speed). Both factors had significant main effects; direction of motion, F(1,42) = 12.3, *p* = .0011, and Speed: F(1,42) = 110.7, *p* < .0001. There was no interactive effect between the two factors, F(1,42)= 0.7, *p* = .41. Figure 6 illustrates these results; clearly, the higher speed tunnel motion constricted more the pupils than the slower one. The contracting motion evoked slightly wider pupil responses than the contracting motion but in a similar manner for both stimuli.

### Expanding or Contracting Circles by Reversed Phi

**Figure 7.**
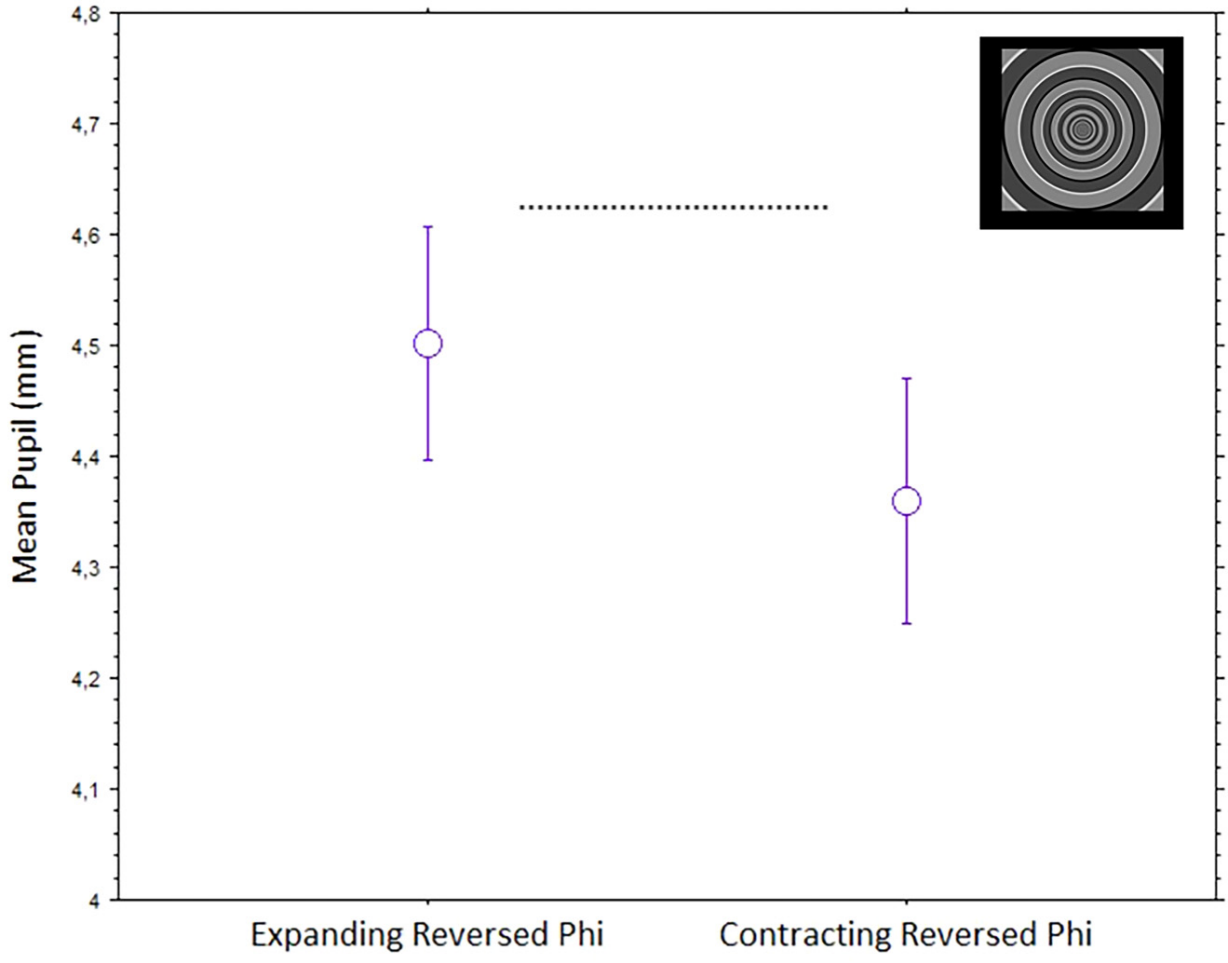
Mean pupils (bars are standard errors) to the dynamic, reversed phi apparent motion, circular tunnels with different directions of motion (expanding and contracting).

One type of stimuli included in the first, dynamic, block included expansive or contracting circular or ring-like patterns where the motion is only suggested by a “reversed phi” or “four-stroke apparent motion.” This gray-level animation yields a perception of expanding or contracting apparent motions of annular patterns, as with the previous stimuli, but with a relatively weakened subjective sense of motion in depth, unlike the tunnel stimuli above. A repeated-measures analysis of variance, with mean pupils (mm) as the dependent variable and Direction of Motion (Expanding/Contracting) as the within-subject factor, revealed a significant effect, F(1,42) = 4.9, *p* = .033. As seen before with the dark spiraling tunnel, the pupils dilated more when viewing an expansive direction of motion than when contracting (Figure 7).

### Two-Dimensional Expanding or Contracting Circles

**Figure 8.**
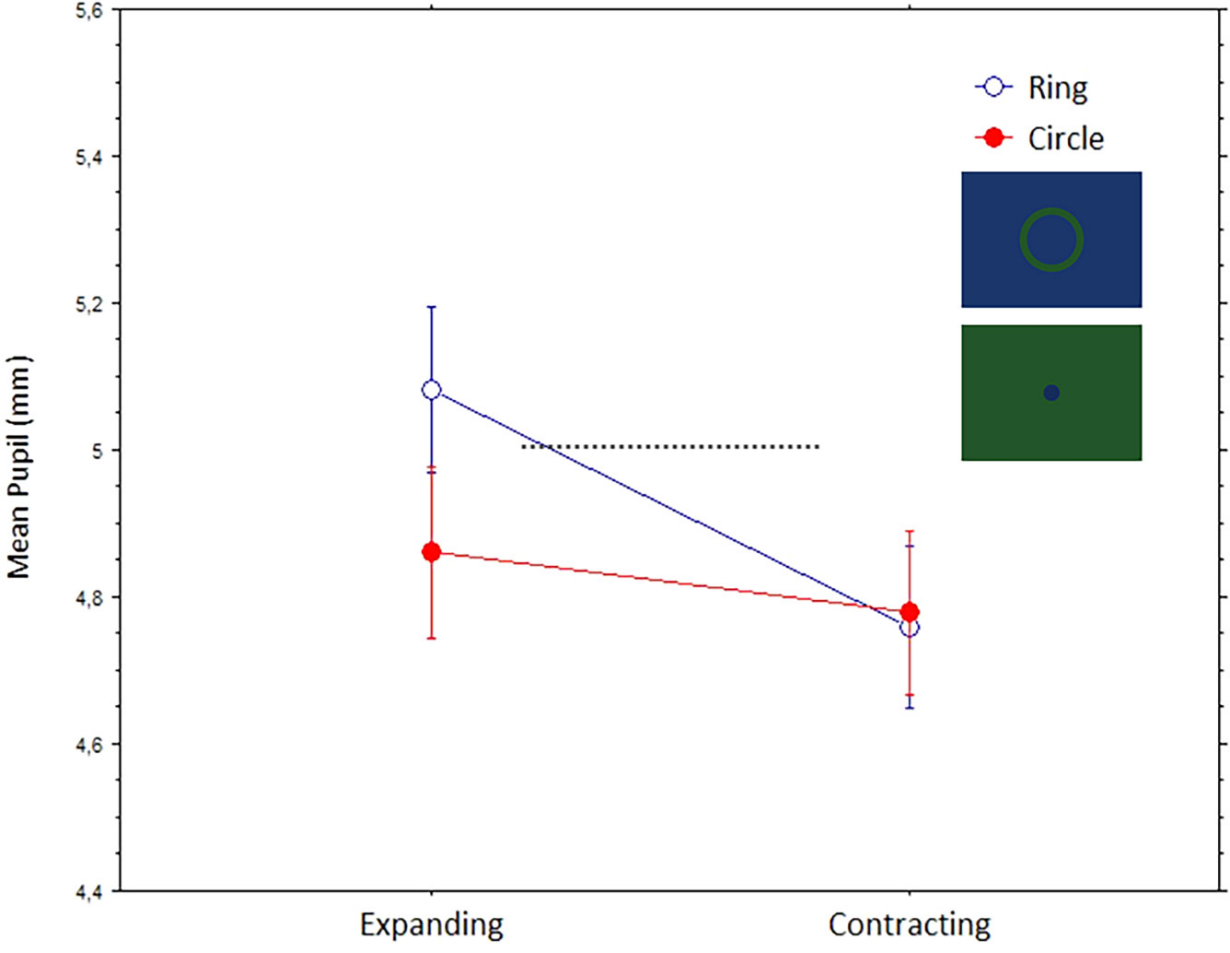
Mean pupils (bars are standard errors) to the dynamic shapes (circle and ring) with different directions of motion (expanding and contracting).

These stimuli consisted of blue/green-colored, rings or circles on an equiluminant green/blue background. A central circle or ring either expanded or contracted, but the motions appeared occurring only in two-dimensional space or over the screen plane. A repeated-measures analysis of variance, with mean pupils (mm) as the dependent variable and Shape (Circle, Ring) and Direction of Motion (Expanding/Contracting) as the within-subject factors, revealed significant effects for Shape, F(1,42) = 17.3, *p* = .0002, and Direction of Motion, F(1,42) = 56.7, *p* < .0001. The two factors interacted, F(1,42) = 56.7, *p* < .0001. Figure 8 illustrates these results; the largest pupil dilation took place when viewing the ring stimuli expanding.

### Subjectively Expanding Dark Holes

**Figure 9.**
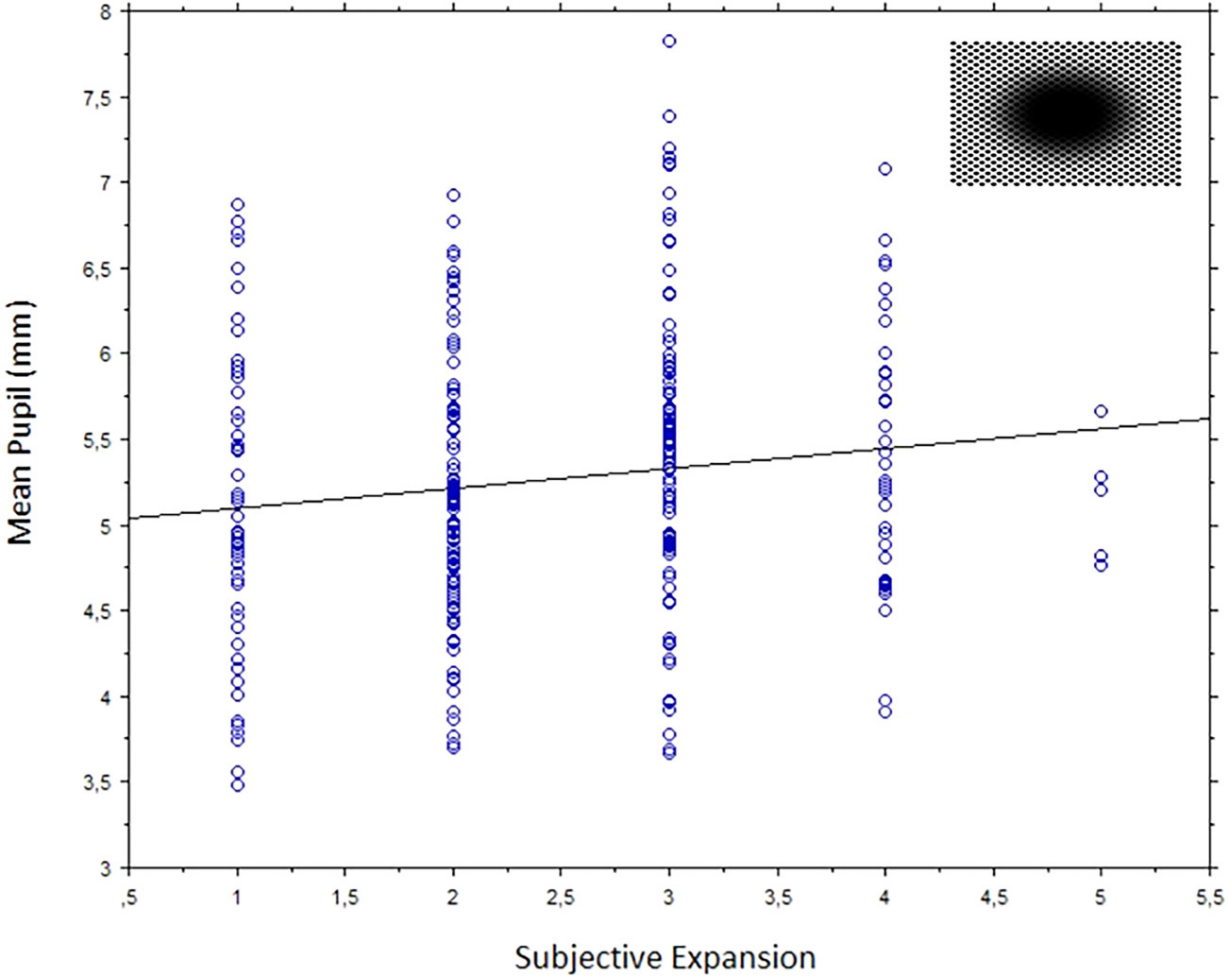
Scatterplot of each participant's mean pupil size (in mm) over the ratings of subject expansion when viewing the expanding dark holes. The gray interpolating line shows the simple regression.

The same participants, except one male participant who could not complete this last block, also viewed illusory stimuli with expanding dark holes, used in our 2022 study. As in that study, participants provided ratings of subjective expansions, right after viewing each stimulus. We performed a linear regression analysis of mean pupils (mm) as the dependent variable and the subjective expansion ratings. This revealed a positive relationship, *R* = .14, slope coefficient = .12, *p* = .016. Figure 9 shows a scatterplot with regression line of these results.

**Figure 10.**
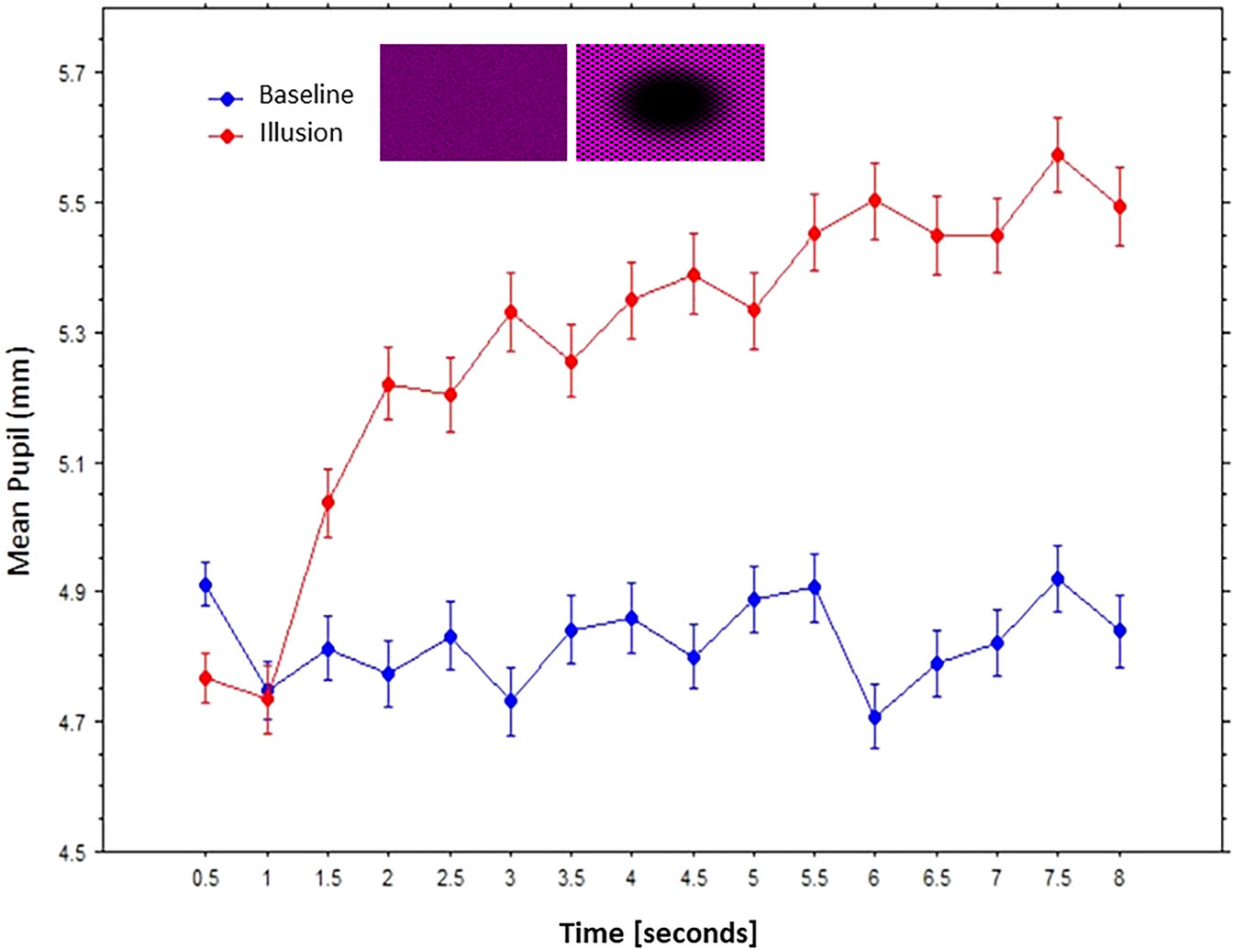
Mean pupils over time in seconds (bars are standard errors) to the illusion (expanding dark holes) and the baseline (scrambled control images).

We also averaged pupil diameters during fixations that occurred from onset and within each consecutive half-second period, yielding sixteen equal epochs, for both the baselines or control stimuli and the “expanding” dark holes. Changes in mean pupils (mm) are shown in Figure 10, which clearly reveals that while pupil enlarged steadily with time when looking at the dark holes, whereas when looking at the scrambled pixels images or control stimuli with matched luminance, the average pupils remained at a low and roughly constant diameter during the same amount of time. In other words, pupil diameters increased over time for the illusions only, and not for the equiluminant control stimuli.

### Relationships Between the Pupil Responses to Optic Flows and to Illusory Dark Holes

Since the same participants viewed the illusory expanding dark holes in one block and the optic flows in another, we assessed whether pupil responses to the static and dynamic illusions are related at the individual level. We performed two separate multiple regression analyses with the pupil responses (in mm) to the optic flows as regressors and the illusory expanding dark holes as the dependent variable. The pupil responses to illusory expanding dark holes were predicted by the individuals’ pupil response to optic flows, but importantly this relationship was significant only with pupil responses to expanding flows with an expanding spiraling motion into a black hole (Table 1) or a linear motion into a dark circular tunnel (Table 2).

**Table 1.** Multiple regression of pupil responses to the expanding dark holes and pupil responses to the spiraling tunnels (expanding or contracting) with different center holes (dark and bright).

	Coefficient	Standard error	Standard coefficient	*t* value	*p* value
Intercept	0.677	0.240	0.677	2.82	.0077
Expanding dark spiral	0.413	0.099	1.32	4.19	.0002
Contracting dark spiral	−0.136	0.107	−0.397	−1.269	.2123
Expanding bright spiral	−0.198	0.129	−0.543	−1.542	.1315
Contracting bright spiral	−0.199	0.128	−0.500	−1.552	.1292

**Table 2.** Multiple regression of pupil responses to the expanding dark holes and pupil responses to the circular tunnels (expanding or contracting) with different speed.

	Coefficient	Standard error	Standard coefficient	*t* value	*p* value
Intercept	0.736	0.234	0.736	3.146	.0033
Expanding tunnel low	0.428	0.185	1.306	2.317	.0261
Expanding tunnel high	−0.405	0.181	−1.081	−2.235	.0315
Contracting tunnel low	0.123	0.154	0.391	0.799	.4295
Contracting tunnel high	−0.252	0.184	−0.721	−1.366	.1802

Finally, we examined the relationship between the pupil dilations to optic flows and the individuals’ subjective ratings of expansion for the illusory expanding dark holes. This last multiple regression analysis showed that the perception of illusory expansion with the dark holes was predicted by the same individuals’ pupil responses to an expanding spiraling motion into a dark hole only (Table 3).

**Table 3.** Multiple regression of subjective expansion (with expanding dark holes) and pupil responses to the spiraling tunnels (expanding or contracting) with different center holes (dark and bright).

	Coefficient	Standard error	Standard coefficient	*t* value	*p* value
Intercept	1.507	0.697	1.507	2.163	.0370
Expanding dark spiral	0.637	0.286	0.807	2.227	.0321
Contracting dark spiral	−0.352	0.310	−0.410	−1.136	.2634
Expanding bright spiral	−0.295	0.373	−0.322	−0.792	.4335
Contracting bright spiral	0.135	0.372	0.135	0.364	.7181

## Discussion

We observed the strongest pupil dilations with stimuli that implied a progressive enlargement of the central pattern, whether this was really happening or just illusory. These pupil dilations were evoked by geometrical shapes that either appeared like virtual-3D tunnels or appeared to have little depth or flat. The effect was especially strong when perceiving a forward motion into a spiraling tunnel and towards a dark center or hole, but it also occurred with a straight motion into a circular dark tunnel. In other words, a common account for many of the observed pupillary effects is that when observers interpret the central patterns as expanding, or as moving forward in depth, the eye pupils adjust by dilating accordingly to the anticipation of increasing darkness. The only exception to this general pupillary response occurred with optic flows in circular tunnels, with alternating brighter and darker “cylindrical” walls, where we observed a relative stronger constriction with expanding motion.

We also found that the pupil responses to the illusory expanding black holes of static patterns were predicted by the individuals’ pupil response to the optic flows, especially when suggesting a spiraling motion or “free fall” into dark tunnel, but not when observers could see a bright center or a “light at the end of the tunnel.” Remarkably, the same individuals’ pupil responses to spiraling motion into a dark tunnel positively predicted the individuals’ sense of illusory expansion with the static, dark hole, patterns. This correspondence across individuals between their pupil responses to both dynamic/static illusory expansive stimuli suggests that all these percepts, despite their input differences, do reflect a common mechanism that derives motion from 2D cues or scenes. Indeed, the susceptibility of each person to one type of illusory perception, either measured by report or the pupil response, was directly related to the observers’ pupil adjustments to the other types of motion perception and to the perceived direction and strength of motion.

An interesting finding was that expansive shapes or motions expanded the pupils. This finding cannot be explained by the eyes’ convergence that takes place when observers attend to objects that are perceived to be near or when the observer moves closer to them. In these cases, the reflexive pupillary response is a constriction (e.g., Kodaka et al., 2003). Interestingly, this happens also when only imagining a known object (e.g., a pen or a car) at an imagined close distance (Sulutvedt et al., 2018). However, pupils dilate when the eyes diverge and focus on distant points in depth or onto numerous and unstructured elements (e.g., Castaldi et al., 2021; Nowack et al., 2024), also when just imagining distance (Sulutvedt et al., 2018). Considering that the present dark tunnels had no visible objects, and suggested a heading motion into a distant emptiness, the present pupil response seems consistent with a “far-reflex” response of the pupil to the illusion of movement into a distant void. In addition, other studies indicate that the pupil constricts to equiluminant gratings of an intermediate-to-high spatial frequency, especially right after a sudden motion onset (e.g., Barbur & Thomson, 1987; Gao et al., 2020; Hu et al., 2019; Sahraie & Barbur, 1997). Such an effect cannot account for the present findings, since we found larger dilations to the higher spatial frequency stimuli than those of low frequency. In the pupillometry literature, there are also hints that pupil sizes tend to match the breadth of the attended area (e.g., Daniels et al., 2012; Nowack et al., 2024; Vilotijević & Mathȏt, 2023); this effect seems consistent to that observed in the present experiment, where pupils were larger with the expansive stimuli compared to those that contracted (e.g., with colored circles and rings).

As mentioned, an exception to the finding of larger dilations with expansive stimuli occurred with the optic flows in circular tunnels, where we observed slightly larger pupils to the contracting stimuli (Figure 6). We can speculate that, since stimuli suddenly entering the visual field from the periphery are more alerting than those leaving the visual field, this featural difference with the other stimuli may have been responsible for some dilation response with the contracting stimuli (in other words, with the illusory backward motion). Interestingly, a study with the flash-lag motion illusion (e.g., Shi & Nijhawan, 2008) showed that a foveopetal or centripetal motion (i.e., towards the point of fixation) yields stronger effects than a fugopetal or centrifugal motion (i.e., away from fixation). Note that this finding is opposite to reports of illusory motion of peripheral static stimuli that show a stronger bias towards seeing centrifugal illusory motion (Zhang et al., 2013). Moreover, as also visible in Figure 6, the pupils constricted relatively more to the stimulus with relatively higher speed. Previous studies have shown inconsistent results about the effect of speed of motion on either dilations (e.g., Wang et al., 2021) or constrictions (e.g., Sahraie & Barbur, 1997). An additional factor to consider is the plane and direction of motion (Beukema et al., 2017), which in most studies occurred laterally (e.g., from left to right on the screen), whereas, in displays with optic flows or self-motion in depth, there is also adjustment in eyes’ vergence, based on the perceived depth of the target of gaze or the perceived distance of the vanishing point (e.g., Frey & Ringach, 2011). As mentioned, Sulutvedt et al. (2018) found that simply imagining a target object as being “seen” at near/far distances resulted in oculomotor adjustments consistent with the near-triad response in perception.

Finally, some considerations about the fact that, in general, these illusions are experienced at the cost of veridicality since the observer is not at all moving, in any direction, into a real space. In the present account, both the illusory motion and the preparatory pupil adjustments represent “optimal” responses that the visual system has learned from repeated experiences (by the individual or even the species) of the natural statistics of the visual world (Changizi et al., 2008; Long et al., 2006). A key role here would be played by internalizing the ecological regularities about light and the risks of sudden increases or decreases in brightness (Suzuki et al., 2019; Zavagno et al., 2017). Though violating the physical measurements, these illusory perceptions may be sustained and robust over repeated observation, because they are inferences based on all past visual experiences. That is, we assume that the likelihood is high that a specific sensory constellation yields a useful prediction of the next event. Indeed, if the next experience is likely to constitute a danger or threat for the observer, as when visibility is impaired by darkness or there is a risk of being dazzled by strong glare (Laeng & Endestad, 2012; Suzuki et al., 2019), these perceptions, as well as their related pupillary adjustments, are both likely to reduce injury or enhance survival. In general, optical illusions of dynamic changes in shape or viewpoint would seem particularly useful, because anticipating such events can divert the costs of injury or extinction. Clearly, the outcomes of motion, either by the observer or by external objects, can carry significant behavioral costs and benefits.
